# Urban Consumer Behaviors in Kochi, India: Food Choice Motivations, Food Label Literacy, and Nutrition Panel Use

**DOI:** 10.1155/ijfo/6568539

**Published:** 2026-04-06

**Authors:** E. Rajashree Bhagyanath, Aswathy Sreedevi, Sydney R. Santos, Renjitha Bhaskaran, Joel Gittelsohn

**Affiliations:** ^1^ Department of Community Medicine and Public Health, Amrita Institute of Medical Sciences and Research Centre, Kochi, Kerala, India; ^2^ Department of International Health, Johns Hopkins Bloomberg School of Public Health, Baltimore, Maryland, USA, jhu.edu; ^3^ Department of Biostatistics, Amrita Institute of Medical Sciences and Research Centre, Kochi, Kerala, India

**Keywords:** consumers, food choice motivation, food label literacy, nutrition panel use, urban

## Abstract

As consumer preferences shift, a nutritional revolution marked by increased consumption of energy‐dense processed foods is driving a rise in chronic, noncommunicable diseases. Factors influencing food purchasing behaviors were assessed among the urban population of Kochi, India. A community‐based cross‐sectional survey was conducted among 680 participants aged 18–60 years. Health benefits, convenience, and sensory appeal were found to be the most significant factors considered while purchasing packaged food. Participants who were younger (*p* = 0.002), female (*p* = 0.011), and/or had at least one reported comorbidity (*p* = 0.010) were significantly more concerned with the food′s sensory appeal, while participants from lower socioeconomic backgrounds were significantly more likely to consider price (*p* = 0.007). Furthermore, consumers who placed importance on the natural content and health‐promoting aspects of food were significantly more likely to have food label literacy (*p* < 0.05) and/or use nutrition panels (*p* < 0.05). Participants with higher food label literacy were more likely to use nutrition panels (*p* = 0.017, adjusted *R* ^2^: 0.017). These findings underscore the importance of tailoring public health interventions to demographic‐specific motivations. Community‐based educational interventions shown to improve food label literacy and utilization may help promote individual consideration of natural and health‐promoting aspects of food products while shopping, which is associated with healthier food consumption patterns.

## 1. Introduction

Noncommunicable diseases (NCDs) are currently responsible for roughly 63% of all fatalities in India [1], with diet‐related NCDs (DR‐NCDs) on the rise [2]. Obesity in particular is becoming more pervasive; India recorded a significant increase in overweight and obese adult populations [3]. Younger age groups have also experienced this trend, with currently 38 million children aged 5 and under being overweight or obese [4].

The growing prevalence of DR‐NCDs has been linked to India′s nutrition transition, which is characterized by a widespread increase in the intake of energy‐dense and processed foods such as refined grains, fried foods, and snacks with high salt or sugar content [5]. This is also reflected in the growing food and beverage sector, which accounts for 3% of India′s GDP and about two‐thirds of its total retail market [6]. One cause of this nutrition transition is India′s expanding class of urban workers, many of whom rely on the convenience of processed foods for their meals [7].

In recent years, the state of Kerala, India, has experienced both a nutritional and epidemiological transition, contributing to a substantial rise in NCDs, placing it among the Indian states with the highest NCD burden [8, 9]. Dietary factors, particularly unhealthy eating habits, have been identified as a key risk factor in the rising prevalence of NCDs in Kerala [10]. One major consequence of these changing dietary patterns is the increasing prevalence of overweight and obesity, which are important precursors to several DR‐NCDs, including diabetes [11]. In urban Kerala, approximately 40.4% of women and 40.1% of men are overweight or obese, which is comparable to other Indian urban centers such as Chandigarh city (43.9% of women and 34.4% of men) and urban Delhi (41.2% of women and 37.9% of men). The prevalence of diabetes, a major DR‐NCD, is high in Kerala with 24.8% women and 27.4% men having blood sugar levels greater than 140 mg/dL. This prevalence is comparable to that observed in other urban areas, such as Andhra Pradesh, where 23.2% of women and 24.9% of men have blood sugar levels exceeding 140 mg/dL [12]. Dietary and lifestyle transitions that increase NCD risk, such as greater consumption of processed and energy‐dense foods as well as reduced physical activity, are well‐documented among several Indian urban populations, including Kerala [13, 7].

Kochi is the largest urban agglomeration in the state of Kerala [14]. As a Tier‐2 city, as designated by the Government of India [15], Kochi is similar to other Tier‐2 Indian cities such as Chandigarh, Faridabad (Delhi), Visakhapatnam(Andhra Pradesh), and Mysuru (Karnataka) in terms of city′s overall quality of life, social development, population size, economic development, infrastructure, educational institutions, and healthcare facilities [15]. These patterns suggest that Kochi′s urban findings may be broadly comparable to other Indian urban centers.

Intervention focusing on dietary intake as a key risk factor for NCDs is a pressing need across the country [16]. One avenue for targeting food consumption patterns, and ultimately diet‐related health outcomes, is to address food purchasing choices [5]. Pre‐existing research has found that purchasing decisions are the result of a complex process with two types of influencing factors: internal or food effects, such as sensory aspects and physiological preferences, and external or nonfood effects, including psychological, social, and cultural factors [17]. Some of these factors are shaped by the information about the nature and characteristics of food provided on food item labels, which can be used by consumers to make informed decisions about their purchases [18]. However, so far, there has been minimal research on the factors influencing food purchasing choices among Indian consumers. Existing studies have only assessed a small subset of potential factors, primarily those relating to perceived healthfulness and sensory appeal [19, 20]. Furthermore, current literature on this topic has relied on qualitative approaches and has yet to employ quantitative collection methods [19, 21] where associations can be examined statistically. Although previous research in India has investigated food choice motivation factors, food label literacy, and nutrition panel use [22–25], these topics have so far been investigated in isolation. Since there is a significant research gap in quantitatively linking these domains, this study is aimed at uniquely examining how nutrition panel use and food label literacy influence specific food choice motivators such as price, convenience, sensory appeal, natural content, weight control, and health within an urban Indian population. By statistically modeling these relationships, the study offers a data‐driven understanding of how informed consumers translate nutritional information utilization into actual purchasing preferences. This quantitative assessment of the relationship between label engagement and food choice adds novel insight into consumer behavior during India′s ongoing nutrition transition and provides evidence‐based direction for targeted public health and behavioral interventions in urban settings.

### 1.1. Conceptual Framework for Examining Determinants of Food Purchasing

**Figure 1:**
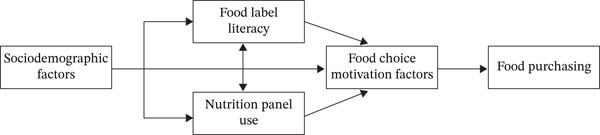
Food purchasing conceptual framework.

The conceptual framework for the present study (Figure 1) was developed by incorporating elements from a conceptual antecedents–decisions–outcomes framework of consumer behavior toward food and nutrition labels [26] and a framework of cognitive processes underlying the use of food labels [27], both proposed in earlier studies. Food choice motivation is influenced by food label literacy and nutrition panel use which in turn are determined by sociodemographic factors such as age, gender, education, and socioeconomic status.

The study is aimed at investigating the following:

1. What is the impact of sociodemographic factors on food choice motivation factors, food label literacy, and nutrition panel use?
2. What is the level of food label literacy and nutrition panel use among urban Indian consumers, and what is their relationship with each other?
3. What is the impact of food label literacy and nutrition panel use on food choice motivation factors?
4. What food choice factors (such as price, sensory appeal, and health benefits) motivate urban consumers in India while purchasing packaged foods?

## 2. Materials and Methods

### 2.1. Study Population

A community‐based cross‐sectional study was conducted in the Kochi Corporation of Ernakulam District in Kerala, India, from March to July 2021. The study population was made up of Kochi Corporation residents aged 18–60 years. The age range of 15–59 years is generally considered the working‐age group in India [28]; however, in this study, we included participants aged 18–60 years to align with the legal age of adulthood and the upper limit of employment age/retirement age [29, 30]. Similar age ranges have been utilized in recent Indian‐based studies on food label literacy and nutrition panel use, as well as previous research on consumer behavior [19, 31–35].

Additionally, participants must have purchased at least one labeled and packaged food product in the last 6 months. The study excluded households with people who were unable to read, write, and comprehend English because Indian food labels are written in English and reading food labels was an essential part of study participation. Other exclusion criteria included households which were locked, indicating that these families had relocated to other cities, and households where residents were self‐isolating due to being infected with COVID‐19. Only one person per household was recruited for the study.

### 2.2. Sample Size Estimation

Sample size was calculated based on the research article by Mor and Sethia [22]; the mean and standard deviation, respectively, for the food choice types were found to be 4.90 and 2.32 for price, 5.39 and 2.14 for health, 4.36 and 1.63 for convenience, 6.50 and 0.25 for sensory appeal, 1.93 and 1.26 for natural content, and 3.21 and 2.32 for weight control.

With 95% confidence and 20% allowable error, using the formula *n* = *Z* ^2^/*d* ^2^ *σ* (where *Z* = 1.96, *d* = 20*%* or 0.02, and *σ* is the standard deviation), the minimum sample size comes to 521 (with price), 440 (with health), 257 (with convenience), 154 (natural content), 522 (with weight control), and 6 (sensory appeal).

The sample size taken for the study is the highest among these which is 521. After multiplying by the design effect of 1.3, the minimum sample size is worked out to be 677. The present study has recruited 680 participants.

### 2.3. Sampling Technique

To include samples from all parts of Kochi Corporation, cluster sampling was opted for. Given the variation in population size across clusters (divisions), the probability proportional to the sample size method was applied to select clusters and ensure representativeness.

Kochi Corporation has five administrative zones, subdivided into 74 divisions, which were considered clusters. A list of all divisions with their population was prepared, and the total population of each zone was obtained by summing the population of its divisions. The total number of clusters to be taken was calculated by dividing the final sample size by the number of individuals to be taken from each cluster.

The required final sample size was 677, with 20 individuals to be taken from each cluster, giving a total of 34 clusters. The number of clusters selected from each zone was determined by dividing the total zone population by the total population of Kochi Corporation (633,553) and multiplying by 34, the total number of clusters in the sample (Appendix A).

#### 2.3.1. Selection of Clusters From Each Zone

Sampling interval was calculated using the following formula:

Sampling interval=total population/number of clusters=6335533418,634,/=.

A random number (≤ 18,634) was chosen using a random number table. The first cluster was the division containing the cumulative population nearest to this random number. Subsequent clusters were identified by adding the sampling interval repeatedly (“random number + sampling interval”), continuing zone by zone until 34 clusters were selected (Appendix B).

The clearance from the institutional ethical committee (Amrita Institute of Medical Sciences, Kochi, Kerala, India) was obtained through ECASM‐AIMS‐2021‐099 dated February 16, 2021. Informed written consent was obtained from each study participant. Each participant was asked to fill out the food choice, food label literacy, and nutrition panel use questionnaires.

### 2.4. Data Collection

A total of 810 houses were visited, out of which 130 houses could not be enrolled as they did not meet the eligibility criteria. Data were ultimately collected from respondents representing 680 households. A questionnaire was administered to the participants to assess food choice motivations, food label literacy, and nutrition panel use (File S1).

A standardized food choice questionnaire developed by Steptoe et al. was adapted and used to assess food choice motivations [36]. In total, the questionnaire assesses nine domains: price, sensory appeal, weight control, natural content, convenience, health, mood, ethical concern, and familiarity. In the present study, only the first six of these domains were included, as these were found to be the most relevant within Indian contexts, as reported in a 2010 study [37]. The relative importance of each food choice motivation domain was measured on a 5‐point Likert scale, allowing participants to choose from *strongly disagree* (score = 1) to *strongly agree* (score = 5). The domain score for price was obtained through an assessment of three questions, sensory appeal through four questions, weight control through three questions, natural content through three questions, convenience through five questions, and health through six questions. Scores were calculated based on multiple questions per domain, with the average score representing the domain′s importance. For example, sensory appeal was assessed through four questions on the degree of importance the consumer places on the smell, appearance, texture, and taste while purchasing food, and the responses were averaged to determine the final mean score of the domain.

Food label literacy and nutrition panel use were assessed by structured questionnaires adapted from previously established literature [31, 34, 38, 39]. Food label literacy was assessed by evaluating the number of correct responses to seven questions. Participants were shown the label of a common Indian food product, Dal Bukhara or black gram lentil curry, as a reference for answering the questionnaire. Dal Bukhara was chosen for this study because it is a culturally familiar and nutritionally relevant food item that is widely available across urban Indian markets. It carries a standard nutrition panel with certification symbols, making it well‐suited for evaluating multiple dimensions of label comprehension. Its widespread availability and moderate ingredient profile allow it to simulate average consumer decision‐making scenarios encountered during routine shopping, and it can be reasonably generalized to other packaged products. Using the Dal Bukhara label, participants were asked to identify the serving size, the amount of protein and fiber in one serving, and if the product was safe for consumption by individuals with hypertension. They were also asked to interpret packaging logos, including certification seals and vegetarian‐friendly symbols. Correct answers were given a score of 1, and incorrect answers were given a score of 0, with a maximum score of 7 indicating the highest level of food label literacy.

Nutrition panel use was calculated by summing the responses to questions that assessed participants′ use of 20 common nutrition panel components, including calories, fat, and sodium. This questionnaire used a Likert scale to assess the frequency with which each nutrition panel component was consulted when purchasing packaged food. The scores were 0 for *never*, 1 for *rarely*, 2 for *occasionally*, 3 for *often*, and 4 for *always*. Higher scores indicated a greater use of nutrition panels, with a maximum score of 80 and a minimum score of 0.

### 2.5. Data Analysis

When tested for scale reliability, the food choice motivation questionnaire showed good reliability for price, very good reliability for natural content, sensory appeal, and convenience, and excellent reliability for weight control and health (Table 1). Furthermore, the food label literacy questionnaire showed fair reliability (Cronbach′s alpha 0.542), and the nutrition panel use questionnaire showed excellent reliability (Cronbach′s alpha 0.984).

**Table 1:** Reliability statistics for food choice motivation factors in a standardized survey.

Food choice motivation factor	Cronbach′s alpha
Price	0.696
Natural content	0.881
Sensory appeal	0.883
Convenience	0.896
Weight control	0.909
Health	0.954

Data was analyzed using SPSS Version 21.0. A frequency analysis was done to examine the general characteristics of households participating in the study. An independent sample *t*‐test was applied to determine the association between food choice motivations and sociodemographic variables. A chi‐square test was used to identify the association between sociodemographic variables and both food label literacy and nutrition panel use.

To find the correlation between food label literacy and nutrition panel use, a Pearson correlation coefficient was computed, and its statistical significance was tested using linear regression *t*‐test. Lastly, a binary logistic regression was completed for the factors found to be significant in the chi‐square test. Quantitative variables, including food choice motivation factors, were expressed as mean and standard deviation.

## 3. Results

### 3.1. Study Sample

The study recruited 680 participants with a mean age of 38.16 ± 13.74 years. The majority of participants identified as male (59.9%), above the poverty line (86.4%), had at least a graduate degree (79.4%), lived in a household with at least three other people (87%), and/or were without children 18 years or younger (61.3%). The most common employment status was full time (48.8%). The majority (80%) of participants reported that they were not diagnosed with any common comorbidities, which included diabetes, hypertension, heart disease, dyslipidemia, or cancer. Out of the 136 participants who reported having comorbidity, the most common was diabetes (49.2%), followed by hypertension (44.1%), heart disease (15.4%), dyslipidemia (19.11%), and then cancer (2.9%). Almost half (46.5%) of the participants reported that no other family members had comorbidities in their household (Table 2).

**Table 2:** Descriptive statistics of urban residents from Kochi, India, who completed food choice, food label literacy, and nutrition panel use questionnaires (*n* = 680).

Sociodemographic factor	Categories	Frequency	Percentage (%)
Age categories (in years)	18–25	84	12.4
	26–35	290	42.6
	36–45	121	17.8
	≥ 46	185	27.2
Gender	Male	407	59.9
	Female	273	40.1
Education	Not completed primary school	1	0.1
	1–5 years of schooling	7	1
	Up to 10 years of schooling	11	1.6
	10–12 years of schooling	79	11.6
	Intermediate/diploma	42	6.2
	Graduate	333	49
	Postgraduate	202	29.7
	Ph.D.	5	0.7
Employment status	Unemployed	90	13.2
	Retired	45	6.6
	Homemaker	141	20.7
	Part time employed	72	10.6
	Full time employed	332	48.8
Socioeconomic class	Above poverty line (APL)	538	86.4
	Below poverty line (BPL)	85	13.6
Household size	1 member	9	1.3
	2 members	79	11.6
	3–4 members	417	61.3
	5 or more than 5 members	175	25.7
Children under 18 years old in the house	Yes	263	38.7
Comorbidities	Yes	136	20
Diabetes	Yes	67	9.9
Hypertension	Yes	60	8.8
Heart disease	Yes	21	3.1
Dyslipidemia	Yes	26	3.8
Cancer	Yes	4	0.6
Family members with comorbidities	No	316	46.5
	Yes, one person	257	37.8
	Yes, more than one person	107	15.7

### 3.2. Sociodemographic Impacts on Food Choice Motivational Factors

Multiple linear regression analysis showed statistical significance for the food choice motivational factor price (*R* ^2^ = 0.023) and sensory appeal (*R* ^2^ = 0.089) on various sociodemographic factors. Participants in the “below poverty line” category placed more importance on the price of the packaged food they purchased (*B* = 0.748, *p* = 0.007). Younger participants placed greater importance on the sensory appeal of the food; for each unit decrease in age, the score for sensory appeal increased significantly (*B* = 1.75, *p* = 0.002). Compared to males, females were more likely to prioritize sensory appeal as a food choice motivator (*B* = −1.12, *p* = 0.011). Additionally, participants who had at least one reported comorbidity placed more importance on sensory appeal compared to those without comorbidities (*B* = 1.63, *p* = 0.010) (Table 3).

**Table 3:** Independent determinants of the food choice motivation factors.

	Price		Sensory appeal		Weight control		Natural content		Convenience		Health	
	*B*	*p* value	*B*	*p* value	*B*	*p* value	*B*	*p* value	*B*	*p* value	*B*	*p* value
Age	0.522	0.030	−1.750	**0.002**	−0.709	0.062	−0.438	0.275	−0.474	< 0.010	−1.295	0.145
Gender	−0.098	0.634	−1.120	**0.011**	−0.566	0.085	−0.677	0.052	−1.034	0.052	−1.103	0.108
Comorbidities	−0.509	0.058	1.630	**0.010**	—	—	—	—	—	—	0.448	0.664
Food label literacy	−0.038	0.845	1.270	**0.002**	0.585	0.074	1.048	**0.003**	1.308	**0.013**	1.593	**0.019**
Nutrition panel use	0.176	0.356	0.100	0.804	1.763	**< 0.001**	1.582	**< 0.001**	0.711	0.175	3.314	**< 0.001**
Occupation	0.004	0.984	0.150	0.741	—	—	—	—	—	—	—	—
Education	0.014	0.955	0.620	0.346	−0.534	0.317	−0.382	0.488	—	—	−0.167	0.878
Socioeconomic status	0.748	**0.007**	—	**—**	−0.167	0.377	—	—	—	—	0.271	0.486

*Note:* The significant values are in bold emphasise.

### 3.3. Food Label Literacy and Nutrition Panel Use

The mean score for food label literacy was 3.96 ± 1.37 with the majority (60.4%) of participants scoring ≤ 4, indicating low food label literacy among the study population. In assessing nutrition panel use, the mean score was 22.79 ± 23.64, with over half (55.9%) of participants scoring ≤ 23, reflecting minimal use of nutrition panels (Table 4). There was a mild positive correlation between food label literacy and nutrition panel use (*p* = 0.017, *r* = 0.133, adjusted *R* ^2^: 0.017).

**Table 4:** Distribution of food label literacy score and nutrition panel use score.

**Distribution of food label literacy score**		
**Score (minimum 0, maximum 7)**	**Frequency**	**Percentage (%)**
≤ 4	411	60.4
> 4	269	39.6
**Distribution of the nutrition panel use score**		
**Score (minimum 0, maximum 80)**	**Frequency**	**Percentage (%)**
≤ 23	380	55.9
> 23	300	44.1

### 3.4. Sociodemographic Impacts on Food Label Literacy and Nutrition Panel Use

Multivariate regression analysis revealed that food label literacy was almost two times higher among those with more education, specifically those with a graduate degree or higher (aOR = 1.812 [95% CI]). Similarly, female participants were significantly more likely to use nutrition panels compared to males (aOR = 1.541 [95% CI]) (Table 5).

**Table 5:** Independent determinants of food label literacy and nutrition panel use.

**Independent determinants of food label literacy**			
	**B**	**p value**	**Adjusted OR (95% CI)**
Age	0.009	0.967	1.009 (0.662, 1.537)
Gender	0.05	0.783	1.051 (0.736, 1.501)
Education	0.592	**0.013**	1.812 (1.134, 2.896)
Employment status	−0.184	0.324	0.832 (0.578, 1.198)
Socioeconomic status	0.023	0.928	1.023 (0.621, 1.687)
Comorbidities	0.305	0.218	1.357 (0.835, 2.205)
**Independent determinants of nutrition panel use**			
	**B**	**p value**	**Adjusted OR (95% CI)**
Age	0.145	0.494	1.156 (0.763, 1.753)
Gender	0.432	**0.016**	1.541 (1.085, 2.189)
Education	−0.257	0.254	0.773 (0.497, 1.203)
Employment status	0.072	0.693	1.074 (0.753, 1.532)
Socioeconomic status	0.257	0.304	1.292 (0.793, 2.108)
Comorbidities	−0.277	0.243	0.758 (0.477, 1.206)

*Note:* The bold means that value is statistically significant: *p* < 0.05.

### 3.5. Impact of Food Label Literacy and Nutrition Panel Use on Food Choice Motivation Factors

Multiple linear regression analysis showed statistical significance for the relationship between food label literacy and the food choice motivational factors: natural content (*R* ^2^ = 0.109), health benefits (*R* ^2^ = 0.113), convenience (*R* ^2^ = 0.042), and sensory appeal (*R* ^2^ = 0.089). Similarly, nutrition panel use showed statistically significant correlations with natural content (*R* ^2^ = 0.109), weight control aspects (*R* ^2^ = 0.117), and health benefits (*R* ^2^ = 0.113). Participants with higher food label literacy considered the natural content (*p* = 0.003), health benefits (*p* = 0.019), convenience (*p* = 0.013), and sensory appeal (*p* = 0.002) of food items as significantly more important. Additionally, participants who made greater use of nutrition panels while purchasing packaged foods gave significantly higher importance to weight control aspects (*p* < 0.001), natural content (*p* < 0.001), and health benefits (*p* < 0.001) (Table 3).

### 3.6. Assessment of Food Choice Motivation Factors

Overall, the factors that most commonly motivated consumers while purchasing packaged food were the health benefits (mean score 21.15 ± 6.28), convenience of purchase and preparation (mean score 17.68 ± 4.62), and sensory appeal aspects such as taste, smell, texture, and appearance (mean score 13.87 ± 3.78) (Table 6).

**Table 6:** Mean score distribution of food choice motivation factors.

Domains (minimum and maximum score)	Mean score (standard deviation)
Health (minimum 6, maximum 30)	21.15 (6.28)
Convenience (minimum 5, maximum 25)	17.68 (4.62)
Sensory appeal (minimum 4, maximum 20)	13.87 (3.78)
Price (minimum 3, maximum 15)	10.25 (2.41)
Natural content (minimum 3, maximum 15)	10.05 (3.30)
Weight control (minimum 3, maximum 15)	9.63 (3.16)

## 4. Discussion

This is the first quantitative study to assess the relationship between food choice motivators, food label literacy, and nutrition panel use in influencing Indian consumers′ food purchasing decisions. The study identified sensory appeal and price as the only motivational factors significantly linked to sociodemographic characteristics. Younger participants, females, and those with at least one reported comorbidity were more influenced by sensory appeal. Participants from lower socioeconomic backgrounds prioritized price when shopping. Food label literacy was correlated with education, and higher nutrition panel use was more common among females. Both food label literacy and nutrition panel use were associated with several motivational factors. Notably, consumers with higher food label literacy and nutrition panel use placed greater importance on the natural content and health‐promoting aspects of food products. Food label literacy was also correlated with sensory appeal and convenience. Additionally, those with higher nutrition panel use were more concerned about weight control. Health benefits, convenience, and sensory appeal were the most important factors considered by participants when purchasing packaged food items. Furthermore, consumers with higher food label literacy are more likely to use nutrition panels.

These results substantiate the relationships reflected in the present study′s food purchasing conceptual framework. Specifically, sociodemographic factors, including age, gender, and economic status, were found to have a significant effect on consumers′ food choice motivations. Food label literacy and nutrition panel use had a significantly positive influence on each other, and both independently influenced several food choice motivation factors. Food label and nutrition label use therefore appear to have a more intermediary role in determining purchasing decisions.

The high prevalence of female urban residents in Kerala′s workforce with disposable incomes may explain the strong link between identifying as female and prioritizing sensory appeal [40], as their financial independence enables them to appreciate the esthetic and experiential aspects of food. Pre‐existing literature in other parts of India, including a study conducted in Patna, Bihar, has found a similar correlation between food motivation factors and gender [41]. Furthermore, a study from Vellore found that younger individuals were concerned about the taste of packaged foods [42]. As social media has become more commonly utilized among young consumers, visually appealing foods that are more widely featured and promoted on these platforms have gained popularity [7]. As a result, younger individuals may see food as an experience rather than just sustenance, explaining their preference for sensory appeal [43]. Other countries have also reported similar findings; one study revealed that young consumers in Malaysia identified sensory appeal as one of their top factors influencing food choice motives [44].

The use of food labels and nutrition panels among the study population also aligned with previous research. Studies have already highlighted the link between education and understanding food labels [33, 23]. Food label literacy “refers to an individual′s capacity to obtain, comprehend, and utilize information found on nutrition labels” [45]. However, the use of nutrition labels “is an active process that involves searching out information, evaluating its meaning, and making a decision based on that evaluation” [46]. Thus, behavioral and skill‐related barriers appear to influence nutrition panel use more strongly than educational attainment [47]. This may explain why, in the present study, education was a significant determinant for food label literacy but not for nutrition panel use.

The present study also found that females used nutrition panels at higher rates than males. Women in India often handle meal planning and family health [48, 49]; as a result, they may focus on the nutritional value of food by assessing nutrition panels more than other family or household members. Research from other countries has also found significant relationships between women and nutrition panel use, including studies among consumers in London and Egypt [50, 51]. Similar to the present study, a study conducted in New Delhi and Hyderabad observed that consumers concerned about fat and sugar intake, reflecting an interest in weight control and health, made more use of the nutrition facts panel [21]. Studies from Puducherry and South Delhi also found that consumers who frequently used food labels primarily did so to assess health‐related aspects and for weight reduction [35, 52]. These findings are not unique to India; for example, a study in the United States found that individuals who paid more attention to nutrition information on package fronts were more likely to consume a healthy diet [53]. Overall, future research should include an investigation of the limited use of food labels found in the present study, and whether alternative ways, such as front‐of‐package labeling, are needed to convey nutrition information on food items to make it more accessible for consumers [54–56].

Research conducted in different jurisdictions across India has also found health, sensory appeal, and convenience to be some of the most important food choice motivation factors among consumers. However, this study revealed much stronger preferences compared to previous findings [20, 22, 25]. For example, in a study conducted in Mumbai, which also employed the questionnaire developed by Steptoe et al., investigators found health (3.51 ± 0.73), sensory appeal (3.63 ± 0.77), convenience (3.61 ± 0.82), and weight control (3.17 ± 0.84) to be some of the most important factors [25]. Higher mean values for the food choice motivators in the present study may be caused by social, economic, and cultural differences between Kerala and other Indian states. Specifically, Kerala is a relatively high‐income state with a higher average income, the highest Sustainable Development Goal and Human Development Index scores [57]. These facts could explain the stronger preference for motivators that are correlated with more expensive food products, such as convenience and sensory appeal. Furthermore, Kerala is the most literate state in the country [57] and has surpassed the national average in gross enrollment ratio for males and females [58]. The state also has the highest prevalence of chronic diseases in the country [59]. This may mean that Kerala′s residents are more educated and aware of diet‐related health issues, leading to greater concern about the impact of food on health and weight control.

This study′s findings can help guide future interventions for Indian consumers. Since sensory appeal matters to younger individuals and those with comorbidities, promoting healthier food choices in these groups is essential. Schools could include food label education in nutrition curricula to encourage early awareness. Previous studies in India and Brazil were successful in increasing the awareness and use of food labels among students through implementing educational interventions in schools [60, 55]. Similarly, health professionals, nutritionists, and community health workers could also include this knowledge in their nutrition counseling for patients with comorbidities. Multiple studies from various countries, including India, have found positive improvements in consumers′ use of nutrition labels following health education interventions [61, 62]. Furthermore, a study among Latinos in the United States demonstrated that food label use increased significantly after community health workers delivered nutrition education sessions focused on food labels to individuals with Type 2 diabetes [63]. On a more systemic level, it is also important for the food industry to consider improving the sensory aspects of healthy food so that they are more appealing to consumers.

Furthermore, as this study has found that men are less likely to use nutrition panels, measures that target this half of the population are necessary. Future research to assess the feasibility of interventions in different physical and virtual spaces that men tend to congregate is necessary. Several studies have shown how workplace interventions can be successful in promoting food label usage to make healthier decisions [64]. Social media campaigns incorporating imagery and/or messaging directed at men are another potential avenue to be explored, given that the majority of consumers are influenced by food labeling information disseminated through media such as television, newspapers, advertisements, and social media platforms [39, 65]. However, it is worth noting that previous educational interventions that included all genders have still resulted in a positive change in the knowledge of nutrition labeling, attitudes, and behaviors of men, and therefore, interventions specifically for one gender may not be necessary [61].

Leveraging technology can further enhance consumers′ ability to interpret and use nutrition information effectively. International nutrition scanner apps such as Yuka, MyFitnessPal, and FoodSwitch are also available in India, enabling consumers to assess the nutritional quality, additives, and organic status of foods while shopping and to find healthier alternatives [66, 67]. The FoodSwitch app, which was developed by the George Institute for Global Health, provides traffic‐light nutrition ratings to help consumers make quick comparisons between packaged foods. However, its database coverage is still limited to selected product categories in India and may not include all locally available brands, which can restrict its utility for diverse Indian markets [68]. Apps such as TruthIn, Chuki, and FactsScan are developed in India and cover a wider database and include even tackling the problem of misleading information on the labels [69]. These initiatives demonstrate how technology can translate complex nutrition panel data into accessible, actionable information at the point of purchase. Future research could evaluate the effectiveness of such digital tools in improving consumers′ understanding of nutrition labels and influencing healthier food choices.

Public health campaigns that simplify nutrition information can complement educational interventions by making label use more practical and accessible in everyday contexts. The “Label Padhega India” (“India will read labels”) movement was launched in 2024 to help empower consumers and included resources to help consumers understand food labels [70]. “Har Label Kuch Kehta Hai” (every label speaks) campaign launched by FSSAI in 2025 provides guidelines on interpreting labels, including information on FSSAI logos and nutrition panels [71]. These initiatives are aimed at enhancing consumer awareness and encouraging healthier food choices. However, their impact and effectiveness are yet to be evaluated.

Cultural, social, and socioeconomic factors in India play a defining role in shaping how consumers perceive, interpret, and use food labels. India′s food environment is complex, where traditional eating patterns coexist with an expanding market for processed foods driven by urbanization and changing lifestyles [5, 7]. The social structure, in which household food decisions are often made collectively and primarily influenced by women, affects individual engagement with label information [48, 49]. The present study′s finding that female participants were guided by sensory appeal yet had a greater utilization of nutrition panels suggests a dual influence: While taste and appearance remain important drivers, women′s active role in family food provisioning may also heighten their attentiveness to nutritional information and health considerations. Socioeconomic stratification also plays a critical role, as households from higher income groups increasingly prefer convenience‐oriented and sensory‐appealing foods, while lower income consumers remain guided by affordability and price sensitivity [5]. Consistent with this pattern, the present study found that participants from lower socioeconomic backgrounds prioritized price when purchasing foods, highlighting how economic constraints continue to play a dominant role in shaping everyday food choices over sensory or convenience considerations. Furthermore, the predominance of English language labeling presents a barrier in a multilingual country where functional literacy in English remains uneven across regions and socioeconomic groups [72, 73]. Media exposure, marketing of “health” foods, and aspirational consumption patterns among urban middle‐class households in India have also been found to shape perceptions of packaged food quality and healthfulness [7]. Taken together, these findings highlight that food label use in India is not merely an individual behavior but one embedded within cultural, social, and economic contexts. Understanding these contextual influences is crucial for developing interventions that resonate with India′s socially and economically diverse consumer base.

One limitation of the study is that three domains—familiarity, mood, and ethical concern—were not included in the food choice motivator assessment [37]. However, based on previous research, it is unlikely that these are important criteria for this study′s population. Another limitation of this study is that comorbidities were self‐reported and not cross‐checked with evidenced records, although the validity of self‐reported morbidity has been established in previous studies [74]. Furthermore, the eligibility requirement of being able to read English may have introduced selection bias, as individuals with greater English proficiency may differ in their health literacy, dietary habits, and/or purchasing behaviors compared to the general population of Kochi. Future research could explore the experiences of non‐English readers to provide a more comprehensive understanding of food label interpretation across diverse populations, as well as assess how language barriers impact food choices and dietary behaviors. There are inherent limitations related to the sample size. While the number of participants is sufficient to detect general trends and provide reliable insights at the population level, the sample may not be fully representative of all subgroups within the population. As a result, the findings may have limited generalizability, and variations across specific strata such as age groups, gender, socioeconomic status, or educational levels may not be fully captured. Additionally, the study population is limited to urban Kochi, which differs in sociodemographic characteristics, dietary habits, and food purchasing behaviors from other urban areas in India. Therefore, while the findings contribute valuable evidence on nutrition label comprehension and consumer decision‐making, the results should be interpreted with discretion when applying them to other populations or regions.

## 5. Conclusion

Food choice motivation, food label literacy, and nutrition panel use are closely related to food purchasing decisions among Indian consumers. Food label literacy and nutrition panel use, which include information on the natural content and health benefits of food items, enable consumers to make informed choices about their food shopping. Therefore, promoting both may have significant benefits for consumers′ health and well‐being. Concerted campaigns aimed at promoting food label literacy and nutrition panel use can play a crucial role in educating Indian consumers. Furthermore, future research is necessary to explore the discrepancies in gender, age, and education on food choice motivations, label literacy, and nutrition panel use.

## Funding

No funding was received for this manuscript.

## Conflicts of Interest

The authors declare no conflicts of interest.

## Supporting information


**Supporting Information** Additional supporting information can be found online in the Supporting Information section. File S1: The questionnaire used to interview participants is organized into four sections, from Part A to Part D. Part A: Sociodemographic and general details of the respondents. Part B: Standardized food choice questionnaire to assess food choice motivations—price, sensory appeal, weight control, natural content, convenience, and health. Part C: Structured questionnaire to assess food label literacy among the participants. Part D: Structured questionnaire to assess nutrition panel use among the participants.

## Data Availability

The data that support the findings of this study are available on request from the corresponding author. The data are not publicly available due to privacy or ethical restrictions.

## Appendix 1

**Table A1:** Number of clusters required from each zone.

Sl. no.	Zone	Number of divisions	Total population	Number of clusters
1.	Edapally	15	133,146	7
2.	Vyttila	14	109,789	6
3.	Central and Pachalam	15	124,620	7
4.	Fort Kochi and Mattancherry	20	168,591	9
5.	Palluruthy	10	97,407	5
Total		74	633,553	34

## Appendix 2

**Table A2:** Divisions selected for survey using probability proportional to sample size sampling.

Zone	Division no.	Division name	Total population	Cumulative population	Identified cluster	Cluster no.
Edapally	33	Elamakkara North	8547	8547		
	34	Puthukalavattom	9034	17,581	13,981	1
	35	Ponekkara	8757	26,338		
	36	Kunnumpuram	7589	33,927	32,615	2
	37	Edapally	9183	43,110		
	38	Devankulangara	8634	51,744	51,249	3
	39	Karukappilli	7638	59,382		
	40	Mamangalam	5770	65,152	69,883	4
	41	Padivattom	11,745	76,897		
	42	Vennala	9708	86,605	88,517	5
	43	Palarivattom	9354	95,959		
	44	Karanakodam	8582	104,541	107,151	6
	70	Kaloor North	8968	113,509		
	71	Elamakkara	7881	121,390	125,785	7
	72	Pottakuzhi	11,756	133,146		
Vyttila	45	Thammanam	9938	9938	11,390	1
	46	Chakkaraparambu	6557	16,495		
	47	Chalikkavattom	4952	21,447		
	48	Ponnurunni East	9126	30,573	30,024	2
	49	Vyttila	7524	38,097		
	50	Chambakkara	8024	46,121	48,658	3
	51	Poonithura	8935	55,056		
	52	Vyttila Janatha	10,726	65,782	67,292	4
	53	Ponnurunni	5119	70,901		
	54	Elamkulam	6279	77,180		
	55	Girinagar	11,267	88,447	85,926	5
	56	Panampilly Nagar	7933	96,380		
	57	Kadavanthra	5522	101,902	104,560	6
	63	Gandhinagar	7887	109,789		
Central and Pachalam	31	Vaduthala West	9025	9025		
	32	Vaduthala East	7795	16,892	14,150	1
	58	Konthuruthy	9973	26,793		
	59	Thevara	4569	31,362	32,784	2
	60	Perumanoor	5941	37,303		
	61	Ravipuram	8721	46,024		
	62	Ernakulam South	10,047	56,071	51,418	3
	64	Kathrikadavu	8734	64,805		
	65	Kaloor South	4414	69,219	70,052	4
	66	Ernakulam Central	5970	75,189		
	67	Ernakulam North	9018	84,207	88,686	5
	68	Ayyappankavu	10,174	94,381		
	69	Thrikkanarvattom	10,488	104,369	107,320	6
	73	Pachalam	8352	113,221		
	74	Thattazham	11,399	124,620	125,954	7
Fort Kochi and Mattancherry	1	Fort Kochi	10,279	10,279		
	2	Kalvathy	7814	18,093	17,519	1
	3	Earaveli	6425	24,518		
	4	Karippalam	8882	33,400	36,153	2
	22	Mundamveli	14,723	48,123		
	23	Manassery	8403	56,526	54,787	3
	24	Moolamkuzhi	8121	64,647		
	26	Nazarat	7277	71,924	73,421	4
	27	Fort Kochi Veli	7456	79,380		
	28	Amaravathy	9439	88,819	92,055	5
	7	Cheralai	7741	113,812	110,689	6
	8	Panayappilly	11,429	125,241		
	9	Chakkamadam	5046	130,287	129,323	7
	10	Karuvelipady	7673	137,960		
	11	Thoppumpady	9266	147,226	147,957	8
	25	Chullikkal	6623	153,849		
	29	Island North	4666	158,515		
	30	Island South	10,076	168,591	166,591	9
Palluruthy	12	Tharebhagom	8925	8925	11,873	1
	13	Kadebhagam	10,203	19,128		
	14	Thazhappu	10,699	29,827	30,507	2
	15	Edakochi North	9574	39,401		
	16	Edakochi South	7808	47,209	49,141	3
	17	Perumpadappu	9442	56,651		
	18	Konam	9863	66,514	67,775	4
	19	Palluruthy Kacheripady	11,100	77,614		
	20	Nambiapuram	8747	86,361	86,409	5
	21	Pullardesam	11,046	97,407		
