# Supplementary material for: Urban Consumer Behaviors in Kochi, India: Food Choice Motivations, Food Label Literacy, and Nutrition Panel Use
Source: Int J Food Sci. 2026 Apr 6;2026:6568539. doi: 10.1155/ijfo/6568539 (PMC13053941; doi:10.1155/ijfo/6568539)
Supplement: Supplementary file 1 — Supporting Information Additional supporting information can be found online in the Supporting Information section. File S1: The questionnaire used to interview participants is organized into four sections, from Part A to Part D. Part A: Sociodemographic and general details of the respondents. Part B: Standardized food choice questionnaire to assess food choice motivations—price, sensory appeal, weight control, natural content, convenience, and health. Part C: Structured questionnaire to assess food label literacy among the participants. Part D: Structured questionnaire to assess nutrition panel use among the participants. [file IJFO-2026-6568539-s001.docx]

**SUPPLEMENTARY FILE 1**

**QUESTIONNAIRE**

**PART A- Sociodemographic and other details**

1. Age - _______

2. Gender

1. Male C Other
2. Female D Prefer not to say

3. Household size (including participant)

1. 1 Member C 3-4 members
2. 2 Members D 5 or more than 5 members

4. Colour of your ration card

1. Yellow card (BPL) C Blue card (APL)
2. Pink card (BPL) D White card (APL)

5. Education of the respondent

1. Ph. D F. Middle school certificate
2. Postgraduate G. Primary school certificate
3. Graduate H. Not completed primary school
4. Intermediate/ Diploma I. Illiterate
5. High school certificate

6. Which of the following best describes your employment status?

1. Full time employed D. Homemaker/ housewife
2. Part time E. Retired
3. Unemployed

7. Are there children under 18 years in the house?

1. Yes
2. No

8.. Has a doctor or other health care provider ever told you that you have any of the following?

1. Diabetes. (high blood sugar)
2. Hypertension, (high blood pressure)
3. Cardiac disease, (heart disease)
4. Dyslipidaemia (high cholesterol)
5. Cancer,
6. None

9.Does anyone else (at least 1 other than participant) in the family have any of the same?

1. No
2. Yes, one person
3. Yes, more than one more person

**PART B- Food Choice**

1. How much do you agree with the following-

It is important to me that the packaged food I buy -

| **Food choice factor** | **Question** | **Strongly disagree** | **Disagree** | **Neutral** | **Agree** | **Strongly agree** |
| --- | --- | --- | --- | --- | --- | --- |
| Price | 1. is not expensive |  |  |  |  |  |
|  | 2. is cheap |  |  |  |  |  |
|  | 3.is of good value for money |  |  |  |  |  |
| Sensory appeal | 4.smells nice |  |  |  |  |  |
|  | 5.looks nice |  |  |  |  |  |
|  | 6. has a pleasant texture |  |  |  |  |  |
|  | 7.is one that tastes good |  |  |  |  |  |
| Weight control | 8. is low in calories |  |  |  |  |  |
|  | 9. is one that helps me control my weight |  |  |  |  |  |
|  | 10.is low in fat |  |  |  |  |  |
| Natural content | 11. contains no additives (food thickening agents, preservatives etc.) |  |  |  |  |  |
|  | 12. contains natural ingredients |  |  |  |  |  |
|  | 13. contains no artificial ingredients (artificial flavour, artificial colour etc.) |  |  |  |  |  |
| Convenience | 14. is one that is easy to prepare |  |  |  |  |  |
|  | 15.can be cooked very simply |  |  |  |  |  |
|  | 16. takes no time to prepare |  |  |  |  |  |
|  | 17.can be bought in shops close to where I live or work |  |  |  |  |  |
|  | 18. is easily available in shops and supermarkets |  |  |  |  |  |
| Health | 19. contains a lot a vitamins and minerals |  |  |  |  |  |
|  | 20. is one that keeps me healthy |  |  |  |  |  |
|  | 21 is nutritious |  |  |  |  |  |
|  | 22.is high in protein |  |  |  |  |  |
|  | 23. is good for my skin/teeth/hair/nails etc. |  |  |  |  |  |
|  | 24 is high in fiber and roughage |  |  |  |  |  |

**Part C- Nutrition Label Literacy**

Use the label below to answer the questions that follow


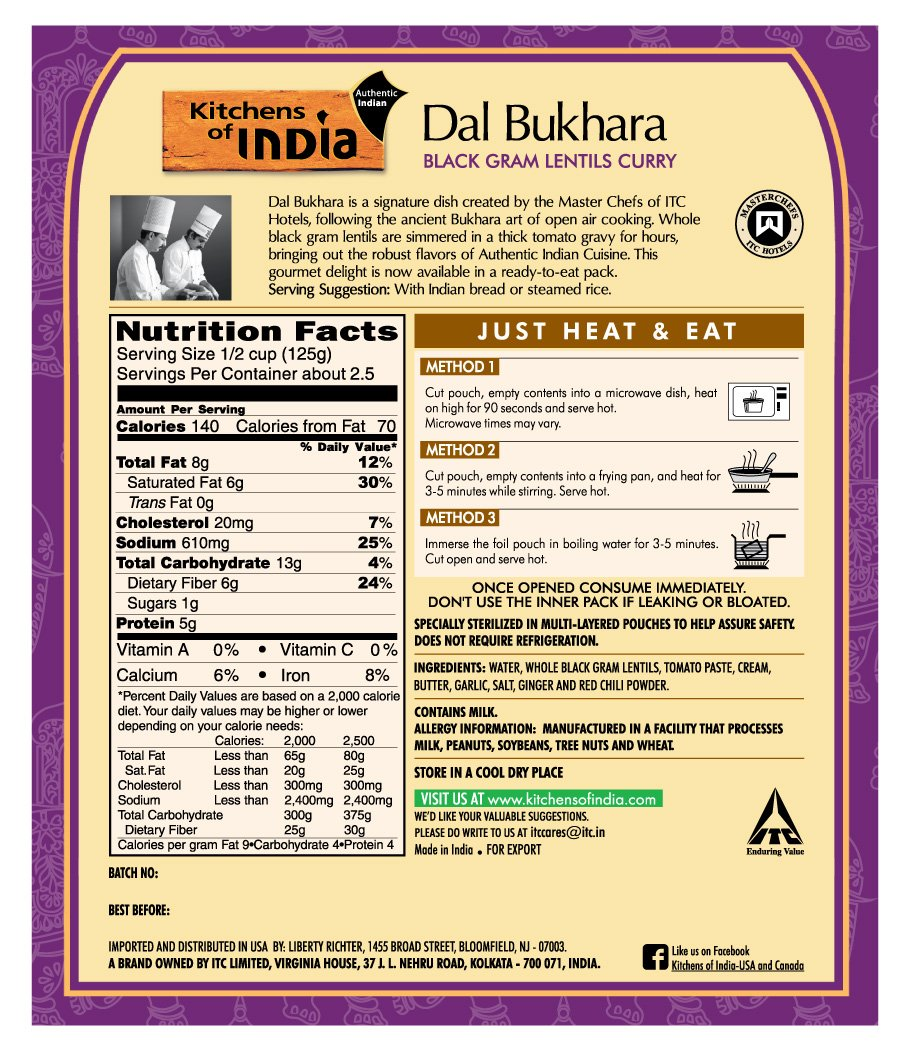


1. What does the term ‘serving size' on the label mean?

1. The amount of this food that nutrition experts recommend that most people should eat
2. The amount of this food most people usually/typically eat at one time
3. The amount of this food present in the whole container
4. I don't know

2. According to this label, one serving size of the product refers to how many cups?

1. ½ cup C. 2 cups
2. 1 D. I don’t know

3. According to this label, from **one serving** of this food how much **protein** will you be getting?

1. 8g C. 6g
2. 5g D. I don’t know

4: According to this label, how many grams of **fibe**r is there in this **whole container** of Dal Bukhara?

1. 8g C. 24g
2. 12g D. 15g E. I don’t know

5.After reading this label, do you think it is safe for a person with high blood pressure/ (high BP) to consume this food?

1. Yes
2. No
3. I don’t know

6. What does the green dot logo indicate?

1. Quality approved C. Imported
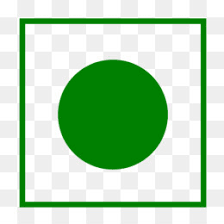

2. Vegetarian D. I don’t know

7.What do these logos indicate regarding the packaged food?


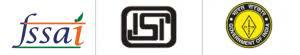


1. Imported from another country
2. Certified food (quality and safety tested)
3. Logos of the manufacturing company

**PART D- Food Label use**

1. How often do you check the following items when reading the food label on food packages?

| Question | Never | Rarely | Occasionally | Often | Always |
| --- | --- | --- | --- | --- | --- |
| a. Serving size |  |  |  |  |  |
| b. Calories |  |  |  |  |  |
| c. Calories from fat |  |  |  |  |  |
| d. Total Fat |  |  |  |  |  |
| e. Saturated fat |  |  |  |  |  |
| f. Trans Fat |  |  |  |  |  |
| g. Cholesterol |  |  |  |  |  |
| h. Sodium |  |  |  |  |  |
| i. Iron |  |  |  |  |  |
| j. Health Claims such as "reduce the risk of heart diseases" |  |  |  |  |  |
| k. Nutritional Claims such as “Low Sodium”, “Sugar free”, ”High fiber” |  |  |  |  |  |
| l. Ingredients list |  |  |  |  |  |
| m. Vitamin A |  |  |  |  |  |
| n. Vitamin C |  |  |  |  |  |
| o. Calcium |  |  |  |  |  |
| p. Total Carbohydrate |  |  |  |  |  |
| q. Dietary Fiber |  |  |  |  |  |
| r. Sugar content |  |  |  |  |  |
| s. Protein |  |  |  |  |  |
| t. Potassium |  |  |  |  |  |
